# Chronic Disease Management for People With Hypertension

**DOI:** 10.3389/ijph.2022.1604452

**Published:** 2022-06-02

**Authors:** Woo-Ri Lee, Ki-Bong Yoo, Jiyun Jeong, Jun Hyuk Koo

**Affiliations:** ^1^ Department of Health Administration, Yonsei University, Wonju, South Korea; ^2^ Institute of Health and Welfare, Yonsei University, Wonju, South Korea; ^3^ Yonsei University Wonju Industry-Academic Cooperation Foundation, Wonju, South Korea

**Keywords:** hypertension, continuity of care, chronic disease management program, difference-in-difference, propensity score matching

## Abstract

**Objectives:** To assess the effectiveness of continuity of care policies by identifying the impact of a chronic disease management program on the continuity of care in patients with hypertension in South Korea.

**Methods:** The propensity score matching method was used to control selection bias, and the difference-in-differences method was used to compare the impact on the treatment and control groups according to the policy intervention.

**Results:** The continuity of care index of hypertensive patients using the difference-in-differences analysis outcome of the chronic disease management program was higher than that of the non-participating hypertensive patients.

**Conclusion:** Continuous treatment is vital for chronic diseases such as hypertension. However, the proportion of those participating in the intervention was low. Encouraging more hypertensive patients to participate in policy intervention through continuous research and expanding the policy to appropriately reflect the increasing number of chronic diseases is necessary.

## Introduction

With a rapidly aging population, major medical issues have emerged as political topics in developed countries. South Korea has the fastest aging population [1] and lowest total fertility rate [2], and therefore needs to be prepared for a growing elderly population. According to the Organization for Economic Cooperation and Development (OECD) Health Statistics (2020), the average health expenditure compared to GDP in 38 OECD countries increased from 7.2% in 2000 to 8.8% in 2018 [3]. In South Korea, the index value was 7.6% lower than the OECD average in 2020. However, considering that it was 3.9% in 2000, South Korea has experienced the steepest increase among the OECD member countries. This is because of the increase in older adults’ medical expenses. Hence, policies to reduce medical costs are required.

An increase in the population of older adults inevitably increases the incidence of chronic diseases. Chronic diseases entail major factors such as poor health, disability, and death. Therefore, the prevention, early detection, and continuous management of diseases are important [4]. Hypertension is one of the most common chronic diseases worldwide. In 2018, 33% of Korean adults over 30 years of age had hypertension [5]. Moreover, hypertension is a significant risk factor for cardiovascular disease and premature death [6]. The enormous socioeconomic and medical costs of chronic diseases can be reduced by preventing disease progression through continuous and systematic management. To do this, an active policy intervention by the government is required.

As of 1 April 2012, the South Korean government implemented a chronic disease management program (CDMP) policy for patients with essential hypertension (ICD 10: I10) and Type-2 diabetes (ICD 10: E11) [7]. The CDMP policy aims to promote health management in primary care by providing continuous care at the same clinics. Information regarding the CDMP policy was provided by a physician and participation in the program was voluntary. CDMPs are applied in hospitals where hypertensive patients wish to continue receiving care. Patients who participate in the CDMP receive a 30%–20% reduction in their copayment for outpatient services. In addition, education on chronic disease management and health counseling services is offered [8]. Previous studies have shown that patients participating in the program have improved adherence to and persistence of drug treatment [9]. Additionally, the risk of complications (acute myocardial infarction, stroke, chronic kidney disease, and heart failure) in patients with hypertension was lower than that in the non-participating group. The cost-effectiveness is also significant [10].

It is necessary to track the change in the continuity of care (COC) of chronically ill patients according to the policy’s purpose of promoting health through the continuous health management of patients. COC is a core attribute of primary care and is a longitudinal and continuous relationship between physicians and patients [11]. Several studies have indicated that improved COC reduces hospitalizations, emergency room visits, medical costs, and risk of death, and increases medication adherence [12–17]. A previous study on the change in medical care continuity due to a CDMP for diabetic patients confirmed that after participation in the program, medical expenses decreased and treatment continuity increased [18].

To date, no study has examined changes in the continuity of treatment due to programs targeting hypertensive patients. Additionally, there are various indicators for measuring COC, sequential continuity of care (SECON), usual provider care (UPC), integrated continuity of care (ICOC), and others, which are rare in studies where multiple indicator values are calculated and compared.

This study evaluated the policy effect in terms of COC by identifying the impact of the intervention of a CDMP on the COC of hypertensive patients.

## Methods

### Data

This study used data from the National Health Insurance Service–National Sample Cohort (NHIS-NSC). The NHI data consisted of medical service claims data of all citizens. As indicated by the NHIS-NSC data, 1 million people (2% of the total population) comprise nationally representative data, stratified into 1,476 according to gender, age, insurance type, and region. Data are randomly sampled according to the stratum [19].

Data from 2010 to 2014 were used in this study. Of the total 1,108,369 participants, 1,051,404 were excluded. Those under 30 were excluded because they had a low prevalence of hypertension (*n* = 361,887). In addition, eligible for medical aid (*n* = 3,781), or deceased (*n* = 39,647) were excluded. Patients who were not diagnosed with hypertension in 2009 were excluded (*n* = 646,089) from the study. 56,965 participants observed between 2010 and 2014 were included in this study.

**FIGURE 1:**
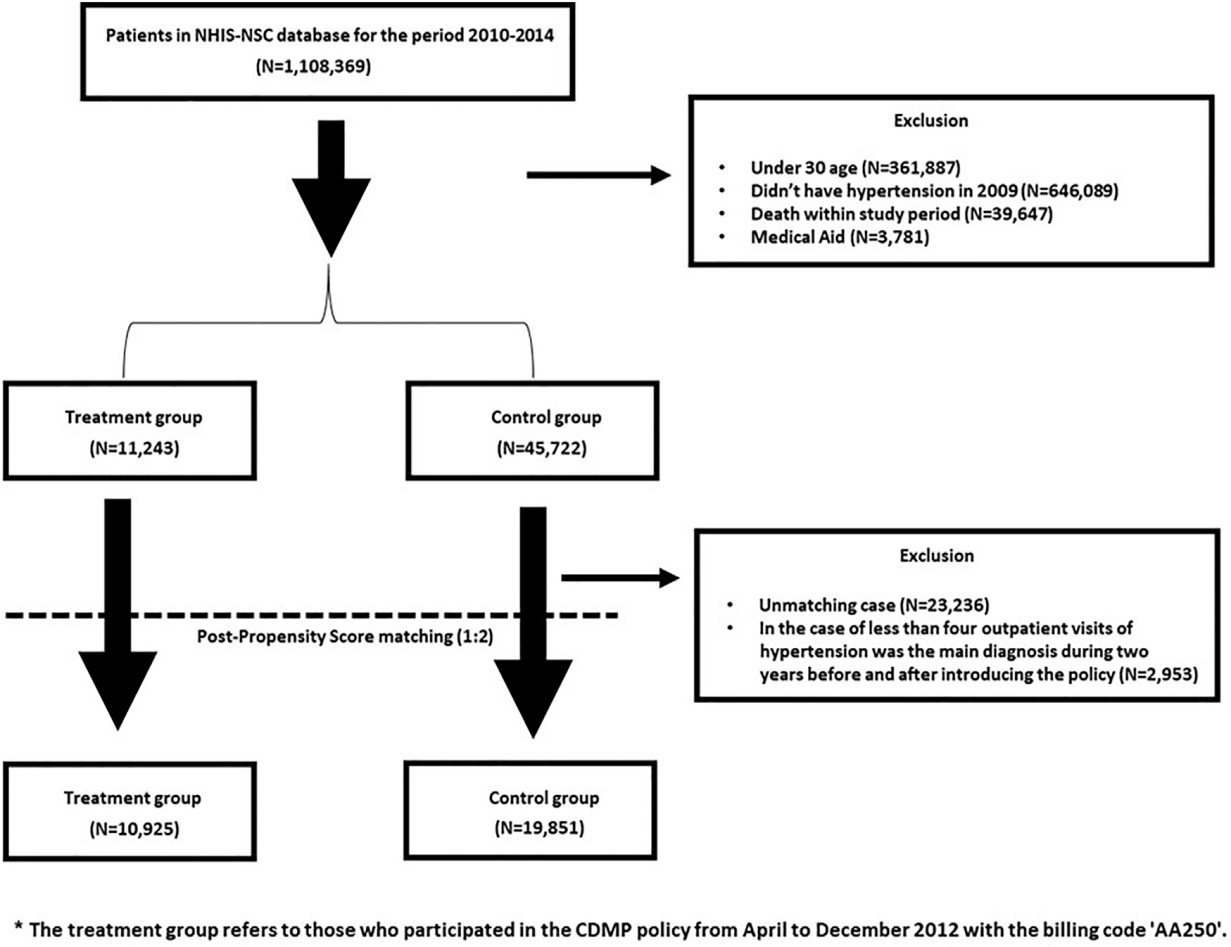
Data flow chart (South Korea, 2010–2014).

Propensity score matching (PSM) was performed before the difference-in-differences (DID) analysis. Among the matched cases, the number of outpatient visits with hypertension as the principal diagnosis was less than four times during each of the 2 years before and after the policy was excluded. The final analysis included 30,776 participants (Figure 1). SAS, version 9.4 (SAS Institute, Cary, NC, United States) was used for the analysis. This study was approved by the Institutional Review Board (IRB) (1041849-202012-SB-184- 01).

### Study Variables

#### Dependent Variable

In previous studies, the COC was calculated on a 1-year basis [12, 18]. According to one study, when the index calculation period was set to 2 years, the probability of hospitalization for the group with a maximum COC score (COC = 1) was lower than when set to 1 year [20]. In other words, if a patient sees the same doctor for a relatively long period, the effect of preventing unnecessary hospitalizations can be improved. In addition, more information can be used if the treatment continuity index is calculated as 2 years. Accordingly, the study was conducted by setting the period for calculating COC to 2 years before and after the policy implementation. Four indicators (COC, SECON, UPC, and ICOC) were used.

In general, the four indicators used in the present study are frequently employed in studies examining COC. Each indicator has its distinct advantages. The UPC helps review the role of primary care by examining the visit rate of regular health care providers [18]. The COC calculates the effect of both the number of health care providers and number of visits [12]. The SECON is relatively easy to calculate and interpret because it is judged based on whether the previous health care provider and current health care provider are the same [12]. Because each treatment continuity index has different characteristics, it is necessary to integrate the COC index [21]. Accordingly, the ICOC, which integrates the above three indicators, was used in this study.

For the policy effectiveness analysis, statistically, we needed to cover only 1 year before and after policy implementation. All indicators have a value between 0 and 1, and the closer they are to 1, the better the COC [12].

The UPC is the number of days of outpatient visits provided by regular health care providers out of all outpatient visits. N is the total number of outpatient visits and $N_{u}$ is the number of outpatient days for regular health care providers [18]. The formula for calculating the UPC is as follows:

$$UPC=\frac{N_{u}}{N}$$ (1)

COC is an indicator used in the absence of a routine health care provider and is considered a more reliable form of measurement, as it is less sensitive to the number of visits by health care providers. N is the total number of outpatient visit days, M is the number of health care providers, and $n_{j}$ is the number of visits to the j th health care provider [20]. The formula for calculating the COC is as follows:

$$COC=\frac{\sum_{j=1}^{M}n_{j}^{2}-N}{N\left(N-1\right)}$$ (2)

The SECON is a measure of the continuity of various health care providers. N is the total number of outpatient visit days and $S_{i}$ is a variable with a value of 1 or 0. If the *i*th medical institution and the i+1st medical institution are the same, it is expressed as 1; otherwise, it is expressed as 0 [12]. The SECON calculation formula is as follows:

$$SECON=\frac{\sum_{i=1}^{N-1}S_{i}}{N-1}$$ (3)

The ICOC combines the above three indicators, and the formula for calculating the ICOC is as follows:

$$ICOC=\left(\beta_{1}UPC+\beta_{2}COC+\beta_{3}SECON\right)/\left(\beta_{1}+\beta_{2}+\beta_{3}\right)$$ (4)

Here, $\beta_{1}$ , $\beta_{2}$ , and $\beta_{3}$ are the first eigenvector values derived as a result of the principal component analysis [12]. The four variables mentioned above have values between 0 and 1 and were used as continuous variables in this study.

#### Independent Variable

Whether or not to participate in the program was classified using claim code “AA250.” When a participant in the CDMP uses outpatient services, the AA250 code is also charged to the prescription, making it possible to distinguish whether or not the patients were participating in the program. Among the participants with underlying hypertension, those who participated in the CDMP in 2012 and who received a prescription under the claim code AA250 were the treatment group. Those who were non-recipients comprised the control group.

#### Control Variable

Age, sex, region, and income were considered individual factors, and disability grade and Charlson Comorbidity Index (CCI) scores were included in the analysis as health-related factors. In the case of age, targets over 30 years of age were coded in units of 5 years and classified up to 65 years of age or above. In the case of regions, Seoul, Gyeonggi-do, and metropolitan cities were regarded as metropolitan cities, and the other regions were regarded as rural. Income was divided into 5 quintiles. Disability is measured on the basis of individual registration and is categorized into physical and mental disability. The disability measurements were subdivided for each disability. The Korean disability classification system divides the grades from 1–6. A grade closer to 1 indicates severe disability and a grade closer to 6 indicates less severe disability. Grades 1–2 indicated severe disability and grades 3–6 represented mild disability [22]. Those without disabilities were classified as non-disabled. The CCI was developed by Charlson in the 1980s as an index to treat a patient’s comorbidity. A total of 17 diseases (e.g., myocardial infarction, dementia, and chronic pulmonary disease) were selected and weighted in the index [23, 24]. This study divided the CCI scores into 0, 1, 2, and ≥3 points.

### Statistical Analysis

The DID method is a commonly used analysis method for examining the effects of a policy. It compares the before and after policy implementation between the treatment group that receives policy benefits and the control group that does not. It can determine the extent to which the treatment group was affected by the policy intervention compared with the control group. In the DID analysis, the effect of the policy can be confirmed through the interaction term between the study group and policy intervention variables [25]. The parallel trend assumption is a strong assumption in this analysis method. The parallel trend assumption is that the trends of the treatment group and the control group before the policy intervention should be parallel, and the result of the DID analysis in a situation where this assumption is not satisfied is not entirely the result of policy intervention [26]. Further, PSM was used to minimize bias when the parallel trend assumption was not satisfied [27]. PSM is based on the probability that an event is an independent variable. The probability corresponding to the treatment group was obtained through a binomial logistic regression analysis. The result was matched with that of the control group at a certain ratio through the derived propensity score. PSM is widely used as an analytical method for determining causal relationships. In our study, the probability of corresponding to the policy beneficiary group was calculated using age, sex, region, income, disability level, and CCI score; 1:2 matching was performed using the greedy matching method. Matching was verified by standardized difference verification to determine whether the control and treatment groups were homogeneous [28].

## Results

Table 1 presents the general characteristics of the study participants. The total number of patients before PSM was 56,965. A total of 11,243 patients participated in the CDMP, and 45,722 participants were control participants. In terms of age, it was confirmed that the number of participants increased as age increased from 25 people aged 30–34 to 2,519 people aged 65 years or older. Regarding gender, there were more women than men and more participants living in large cities. Regarding income, starting with 1,825 people in the first quintile and 3,152 people in the fifth quintile, the number of participants increased as income increased. Regarding disability grade, the number of participants in the program decreased as the degree of disability increased from mild to severe compared with that of non-disabled persons. The CCI score confirmed that the number of program participants decreased as the CCI score increased. Even after PSM, as before, there were more program participants who were older adults, women, living in metropolitan areas, high income, low disability grades, and low CCI scores. The ratio of the treatment to control groups was set at 1:2. When matching the propensity scores, it was confirmed that the two groups were homogeneous because the absolute value of the standardized difference after matching all independent variables was less than 0.1.

**TABLE 1:** General characteristics (South Korea, 2010–2014).

Variable	Classification	Pre-propensity score matching		Post-propensity score matching		
		Control (N = 45,722)	Treatment (N = 11,243)	Control (N = 19,851)	Treatment (N = 10,925)	Standardized Difference
		N (%)	N (%)	N (%)	N (%)	
Age	30–34	147 (85.5)	25 (14.5)	32 (65.3)	17 (34.7)	0.006
	35–39	453 (76.0)	143 (24.0)	234 (63.1)	137 (36.9)	
	40–44	1,383 (74.8)	466 (25.2)	779 (63.2)	453 (36.8)	
	45–49	2,756 (73.1)	1,015 (26.9)	1,815 (64.8)	987 (35.2)	
	50–54	5,141 (71.2)	2,078 (28.8)	3,669 (64.4)	2,028 (35.6)	
	55–59	6,100 (70.3)	2,575 (29.7)	4,538 (64.4)	2,508 (35.6)	
	60–64	5,907 (70.9)	2,422 (29.1)	4,294 (64.6)	2,357 (35.4)	
	≥65	23,835 (90.4)	2,519 (9.6)	4,490 (64.8)	2,438 (35.2)	
Sex	Male	20,318 (79.3)	5,311 (20.7)	9,388 (64.6)	5,149 (35.4)	0.002
	Female	25,404 (81.1)	5,932 (18.9)	10,463 (64.4)	5,776 (35.6)	
Region	Metropolitan	29,626 (78.6)	8,044 (21.4)	14,159 (64.4)	7,820 (35.6)	<0.0001
	Rural	16,096 (83.4)	3,199 (16.6)	5,692 (64.7)	3,105 (35.3)	
Income	Quintile 1	6,973 (79.3)	1,825 (20.7)	3,206 (64.4)	1,769 (35.6)	0.014
	Quintile 2	6,273 (78.0)	1,768 (22.0)	3,167 (64.8)	1,719 (35.2)	
	Quintile 3	6,978 (78.2)	1,947 (21.8)	3,472 (64.8)	1,887 (35.2)	
	Quintile 4	10,093 (79.8)	2,551 (20.2)	4,433 (64.0)	2,491 (36.0)	
	Quintile 5	15,405 (83.0)	3,152 (17.0)	5,573 (64.6)	3,059 (35.4)	
Disability	Non-disabled	40,693 (79.7)	10,384 (20.3)	18,416 (64.6)	10,089 (35.4)	0.012
	Mild	4,543 (85.1)	798 (14.9)	1,351 (63.5)	778 (36.5)	
	Severe	486 (88.8)	61 (11.2)	84 (59.2)	58 (40.8)	
CCI	0	42,864 (80.2)	10,550 (19.8)	18,720 (64.6)	10,245 (35.4)	0.019
	1	1,271 (78.8)	341 (21.2)	565 (62.6)	337 (37.4)	
	2	1,163 (81.0)	272 (19.0)	452 (63.0)	266 (37.0)	
	≥3	424 (84.1)	80 (15.9)	114 (59.7)	77 (40.3)	

CCI, carlson comorbidity index.

**FIGURE 2:**
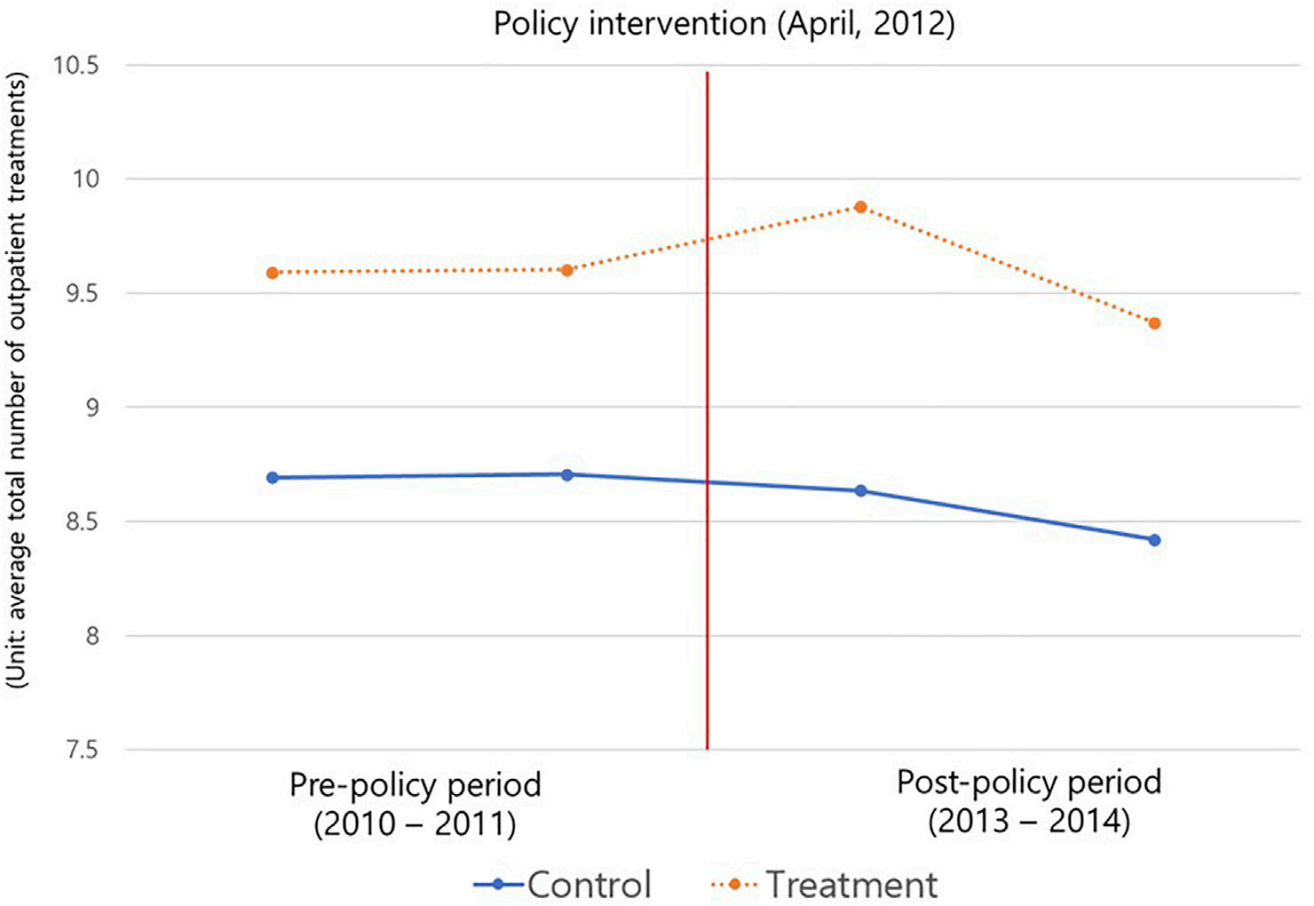
Parallel trend of average total number of outpatient treatments (South Korea, 2010–2014).

In our study, because the COC index was calculated based on the number of outpatient treatments for 2 years, it was confirmed (as shown in Figure 2) that the parallel trend assumption was satisfied through the average total number of outpatient treatments 2 years before and after the policy implementation. Table 2 presents the results of the DID analysis. The effect of the CDMP policy implementation can be confirmed through the treatment × post-policy interaction term. When checking the analysis results for each treatment COC index, for the UPC index, the regression coefficient of the interaction term was not statistically significant at 0.0036 (*p* = 0.089). However, for the ICOC index, the policy recipient’s COC index value after the policy was 0.0041 (*p* = 0.046). The COC indicator was 0.0057 (*p* = 0.049) and the SECON indicator was 0.0028 (*p* = 0.046), indicating that the recipients’ continuity of care after policy implementation was higher than that of non-policy beneficiaries.

**TABLE 2:** Result of difference-in-difference analysis (South Korea, 2010–2014).

Variable	ICOC			COC			SECON			UPC		
	β	SE	*p*-value	β	SE	*p*-value	β	SE	*p*-value	β	SE	*p*-value
Control	Ref	—	—	Ref	—	—	Ref	—	—	Ref	—	—
Treatment	0.068	0.0018	0.001	0.0073	0.0025	0.003	0.0081	0.0013	<0.0001	0.005	0.0018	0.006
Pre-policy	Ref	—	—	Ref	—	—	Ref	—	—	Ref	—	—
Post-policy	0.0166	0.0013	<0.0001	0.0236	0.0018	<0.0001	0.0081	0.0009	<0.0001	0.0168	0.0013	<0.0001
Treatment*Post-policy	0.0041	0.0021	0.046	0.0057	0.0029	0.049	0.0028	0.0014	0.046	0.0036	0.0021	0.089

ICOC, integrated continuity of care; COC, continuity of care; SECON, sequential continuity of care; UPC, usual provider care; β, parameter estimate; SE, standard error; *p*-value, statistically significant; Ref, reference.

## Discussion

### Key Findings

This study used the NHIS-NSC. Among patients with hypertension, the intervention effect of the policy was verified by examining the COC of those who indicated their intention to participate in the CDMP. Those who participated in the program showed higher COC, and the extent of change was greater than those who did not. This indicates that the CDMP had a significant effect on the COC. These results were the same as those of previous studies that confirmed a significant relationship between COC and existing CDMPs [9, 18]. This study analyzed UPC, ICOC, and SECON in addition to the COC indicators, which are typically used as indicators to evaluate the COC according to policy interventions. The validity of the research results was confirmed by providing results from a diverse perspective. Since COC was calculated based on 2 years rather than 1 year, the effectiveness and maintenance of the policy were checked, and the effectiveness of the policy was examined more closely than in a previous study [18]. It was confirmed that the COC significantly increased after the policy intervention across all indicators, except UPC. The group that participated in the program had higher COC than the group that did not.

### Interpretation

The increase in COC in the treatment group indicated that policy intervention is effective in managing chronic diseases that require COC. In previous studies, participation in the program reduced the risk of hypertension complications, and it was suggested that the introduction of the program was effective in terms of the cost required for quality-adjusted life years [8]. The effects of such a policy can be evaluated and predicted using various indicators. When the patient’s COC increased through the program, the aforementioned positive effects were observed. Accordingly, related prior studies also suggested the need for improvement, such as maintaining and increasing indicators through continuous management of COC, indicating the results of the program [29].

When the actual COC increases, consistent and continuous care from the physician affects several secondary outcomes, such as a decrease in avoidable hospitalization [12, 30]. Patients have more discussions with their doctors when they perceive that they have a lasting and deep relationship [31], which helps the doctor better understand the patient’s medical history, personality, and treatment pRef. [32]. Additionally, if the patient’s COC increases, it is possible for physicians to provide higher quality medical services through continuous treatment for the patient. Such long-term trust building between patients and physicians has been reported as a major factor that can reduce medical costs, and the need to observe relevant indicators is increasing [33, 34]. Furthermore, an increase in the COC has been shown to impact health care systems adversely. An increase in chronic diseases leads to increased medical expenses. Therefore, as COC for chronic disease increases, medical costs and length of stay (LOS) also increase, suggesting the need for regular observation of COC [13, 14, 18, 35, 36]. When participating in the program, patients are considered effective in managing their chronic diseases because they are motivated to take care of their diseases and continue to manage them independently. Policies that reduce copayment have been shown to improve medication adherence, and policy beneficiaries are more compliant than non-beneficiaries [37, 38]. Medication adherence also shows a close relationship with COC. There is a need to evaluate and manage effectiveness using various indicators. In previous studies on the effectiveness of other CDMPs, all interventions were effective in improving health condition [39, 40]. However, despite several studies on the effectiveness of the program, the participation rate of the current CDMP is very low, at 12.58% [8]. Therefore, there is an increasing need for research and various activities to increase participation in these programs.

### Strengths and Limitations

This study had several limitations. First, in the case of the COC indicators, it is not possible to consider all factors that may affect the indicators that evaluate the COC within the evaluation period [20]. In other words, setting the period to 2 years is beneficial, as more information can be used. However, the possibility of collecting incorrect information also increases. For example, during the COC measurement period there were unforeseen circumstances such as a patient moving to another region or country for personal reasons or clinic shutdown. This is more likely to occur when measuring COC for 2 years than for 1 year. In this case, COC may show a decline. However, the effectiveness of the system was verified, and the validity of the results was improved using various indicators of COC. Second, although the CDMP policy was implemented in April 2012, program was possible in 2013 and 2014. Of the 2012 policy participants, only those who were also continuously observed in 2013 and 2014 were included. The impact of those who participated in the policy after 2013 was not considered, as it was only considered in 2012, the year the policy came into force. Therefore, future research will require advanced research that includes those who participated in the policy since 2013. Third, owing to the characteristics of health insurance claims data, it was not possible to determine whether non-insured services were provided. For patients who received additional non-insurance services, there may be differences in the quality of care. Thus, further studies should be conducted that consider the provision of non-insured services. Fourth, in this study, the average treatment effect on treated patients was confirmed using the PSM method. Therefore, some participants may have been excluded from the study. Thus, future research is required from the perspective of average treatment effect. Fifth, the policy participation rate is low. We confirmed that the study sample was representative of the national population; however, the participation rate in the program was low because it is not obligatory for medical institutions to solicit participation in the CDMP or to promote the policy.

Despite these limitations, it was confirmed that participation in the CDMP had a significant effect on the continuous health management of patients with hypertension using various treatment continuity indicators. These results can help promote continuous health management of patients through the vitalization of primary care. In addition, the present study used long-term observed nationally representative sample data and controlled bias through the average treatment effect on the treated (ATT).

### Conclusion

This study compared the COC before and after policy intervention to confirm the effectiveness of the CMDP using the NHIS-NSC, which has secured the representativeness of the South Korean population. COC significantly increased after the policy intervention. It was confirmed that the COC of the beneficiary group also increased compared to that of the non-beneficiary group. Continuous treatment is an important care method for chronic diseases, such as hypertension, and an increase in COC positively affects the reduction of future medical expenses. The CDMP policy improved the continuity of treatment which has positive effects such as continuous health management of patients and reduced medical costs. However, the policy participation rate remains low. Despite selecting two representative chronic diseases as the currently targeted diseases, essential hypertension and Type 2 diabetes mellitus, the limitation of not being able to accommodate all chronic diseases remains. Accordingly, incentives are required to encourage participation in policy programs. Additionally, it is necessary to expand the policy by appropriately reflecting the increasing number of chronic diseases through continuous research.

## Data Availability

The data is publicly available in National Health Insurance Sharing Service and can be used after application through the link below. [https://nhiss.nhis.or.kr/bd/ab/bdaba002cv.do].

## Abbreviations

ATT, average treatment effect on the treated; CCI, Charlson comorbidity index; CDMP, chronic disease management program; COC, continuity of care; DID, difference-in-differences; ICD, international classification of diseases; ICOC, integrated continuity of care; IRB, institutional review board; LOS, length of stay; NHIS-NSC, national health insurance service–national sample cohort; OECD, organization for economic cooperation and development; PSM, propensity score matching; SECON, sequential continuity of care; UPC, usual provider care.
